# The association between sarcopenia and diabetic retinopathy: a mediation analysis of inflammatory biomarkers

**DOI:** 10.3389/fendo.2026.1765051

**Published:** 2026-05-21

**Authors:** Fentao Zhong, Hui Cao, Hangju Chen, Baozhen Cao, Mei Tu, Wen Wei, Shihai Li

**Affiliations:** 1 Department of Endocrinology, Longyan First Affiliated Hospital of Fujian Medical University, Longyan, China; 2 The School of Clinical Medicine, Fujian Medical University, Fuzhou, China; 3 Department of Anesthesia, Longyan First Affiliated Hospital of Fujian Medical University, Longyan, China

**Keywords:** blood-cell based inflammatory biomarkers, diabetic retinopathy, mediation analysis, sarcopenia, type 2 diabetes mellitus

## Abstract

**Background:**

Sarcopenia represents a crucial risk factor for diabetic retinopathy (DR). This study is to investigate the association between sarcopenia and DR in patients with type 2 diabetes mellitus (T2DM) and to examine the mediating role of inflammatory biomarkers.

**Methods:**

Sarcopenia was defined according to the Asian Working Group for Sarcopenia 2019. DR was based on ophthalmologic examination. The inflammatory biomarkers included the neutrophil-percentage-to-albumin ratio (NPAR), advanced lung cancer inflammation index (ALI) and pan-immune-inflammation value (PIV). Logistic regression and mediation analyses were conducted to evaluate the associations among sarcopenia, inflammatory biomarkers, and DR and the mediating effects of the biomarkers.

**Results:**

749 participants were included in the analysis. The prevalence of DR was 25.0% and sarcopenia was associated with DR (adjusted odds ratio (*aOR*):1.61, 95% confidence level (*CI*): 1.14-2.26). NPAR, ALI and PIV statistically accounted for this association, explaining 13.44%, 10.41%, and 10.77% of the effect, respectively.

**Conclusions:**

Sarcopenia is an independent associated factor for DR in T2DM. NPAR, ALI and PIV play a partial role in this association. These blood-cell-based inflammatory biomarkers are associated with DR, but their role in clinical screening remains to be determined.

## 1 Introduction

As one of the primary chronic complications of diabetes, diabetic retinopathy (DR) has emerged as the leading cause of visual impairment and blindness among working-age populations globally (1, 2).From 1990 to 2020, the prevalence of blindness attributed to diabetic retinopathy rose by 14.9%, reaching 18.5% (3). Current research has revealed that DR is associated with chronic diseases such as obesity (4), hypertension (5) and dementia (6). Sarcopenia has become not only one of the chronic complications of diabetes but also another key risk factor for DR (7–10).

Multiple epidemiological studies have confirmed a positive association between sarcopenia and DR. In a large-scale cross-sectional study of the Chinese population, Chen et al. further validated this relationship and demonstrated that sarcopenic obesity significantly elevates DR risk (8). In a Japanese cohort, the patients with sarcopenia had a 2.3 times increased risk of DR (9). As for the mechanism, numerous studies have demonstrated that various inflammatory factors play a crucial role in the pathogenesis of sarcopenia. Demir et al. reported higher serum tumor necrosis factor-alpha (TNF-α) levels in sarcopenic cancer patients compared to non-sarcopenic controls (11). Muscle atrophy not only increases inflammatory cytokines (e.g., Interleukin-6 (IL-6), TNF-α) but also activates the nuclear factor kappa-light-chain-enhancer of activated B cells (NF-κB) signaling pathway (12, 13). Some previous studies showed inhibiting these inflammatory cytokines could obtain blood-retinal barrier which may delay the occurrence of DR (14–16).

Accumulating evidence suggests that inflammation plays a pivotal role in the pathogenesis of DR (17–22). A prospective study involving 781 patients with type 2 diabetes showed that elevated C-reactive protein (CRP) was a risk factor for the development of DR (20). Wang et al. found that monocyte-lymphocyte ratio (MLR) was associated with the risk of DR in the NHANES cohort study (21). A study by Atlı et al. involving 120 subjects reported significantly elevated neutrophil and lymphocyte counts in DR patients (22). These clinical findings are corroborated by mechanistic evidence from animal models. Diabetic animal models show increased retinal leukostasis, which contributes to microvascular dysfunction and disruption of the blood–retinal barrier (18). Elevated TNF-α and glycated albumin further amplify inflammatory responses in the retina, thereby promoting the development and progression of DR (23, 24). Acting in concert with various inflammatory factors, glycated albumin collectively drives the initiation and progression of diabetic retinopathy. Collectively, these findings highlight the critical contribution of inflammation to DR development and progression.

Given that chronic low-grade inflammation has also been implicated in the pathogenesis of sarcopenia, inflammation may represent a potential biological link between sarcopenia and DR. Recently, some composite inflammatory biomarkers such as neutrophil-percentage-to-albumin ratio (NPAR), advanced lung cancer inflammation index (ALI) and pan-immune-inflammation value (PIV) have been widely proposed in chronic diseases such as chronic kidney disease, nonalcoholic fatty liver disease and osteoporosis to reflect the inflammatory states of patients (25–27). In a cross-sectional study, after adjusting for all potential confounding factors, NPAR remained significantly associated with the risk of sarcopenia among individuals with arthritis (28). Meanwhile, NPAR has been positively associated with both cardiovascular mortality (29) and diabetic kidney disease (30) among individuals with diabetes. A recent study by He et al. demonstrated that higher NPAR levels remained independently associated with DR risk even after adjusting for various confounders (31). Like NPAR, ALI incorporates neutrophil and albumin parameters, ALI may also be indicative of neutrophil activation and glycated albumin formation during the progression of DR. We hypothesize a potential association between ALI and DR. As PIV has been associated with diabetic nephropathy (32), and specifically, higher PIV levels correlate with increased urinary albumin excretion and declining renal function, reflecting the severity of diabetic nephropathy. Given that diabetic nephropathy shares similar microvascular injury and endothelial dysfunction with DR, PIV may also be associated with DR. However, no study has investigated whether NPAR, ALI and PIV account for the relationship between sarcopenia and DR.

As noted above, this study aims to explore the relationships among sarcopenia, inflammatory biomarkers (e.g., NPAR, ALI, PIV), and DR, and to investigate whether inflammation explain part of the association between sarcopenia and DR.

## 2 Methods

### 2.1 Study population

This cross-sectional study included patients with type 2 diabetes mellitus (T2DM) (age ≥ 18 years) who were admitted to the National Metabolic Management Center at Longyan First Affiliated Hospital of Fujian Medical University in Fujian, China, between July 2023 and June 2024. The Institutional Ethics Research Committee of Longyan First Affiliated Hospital of Fujian Medical University gave its approval to the study (IRB: LYREC 2023-014-01) and the research adhered to ethical guidelines from the relevant research ethics committees. All patients gave written informed consent to take part in the study. The diagnosis of T2DM was defined according to the American Diabetes Association (ADA). Participants were excluded if they met the following criteria (1): diagnosis of other types of diabetes (2); presence of acute complications such as diabetic ketoacidosis, hyperosmolar status, or acute infection (3); presence of severe infection (4); presence of liver cirrhosis, end-stage kidney disease, undergoing renal replacement therapy, or post-renal transplantation (5); pregnancy or lactation (6); presence of malignant tumor or life expectancy of less than 1 year (7); without undergoing a fundus examination or a sarcopenia assessment. Finally, 749 patients were enrolled. A total of 1024 patients were initially screened. Of these, 275 were excluded based on the following criteria: pregnancy or lactation (*n =* 14), other types of diabetes(*n* = 12), acute complications or severe infection (*n =* 19), severe diseases such us end-stage kidney disease, liver cirrhosis or malignant tumor(*n* = 27) and absence of fundus examination (*n* = 38) or sarcopenia diagnostic assessment (*n =* 165). The remaining 749 patients were included in the final analysis.

### 2.2 Data collection and clinical definition

Trained investigators conducted face-to-face interviews using a structured questionnaire designed for this study to collect demographic characteristics and health-related information, including age, gender, smoking history, alcohol consumption, and history of diagnosed diseases. Self-reported health conditions comprised physician-diagnosed hypertension and cardiovascular disease (CVD) and dyslipidemia. After hospitalization, a nurse with five years of clinical experience measured patients’ height, weight, and blood pressure using standardized documentation forms. Venous blood samples were collected in the early morning after an overnight fast of at least 8 hours. All laboratory parameters, including glycosylated hemoglobin (HbA1c), fasting blood glucose (FBG), albumin, and complete blood count, were measured at enrollment using fresh blood samples. Clinical characteristics and biochemical data were subsequently retrieved from the electronic medical record system.

Smoking status was grouped into either “no current smoker” or “current smoker”. Likewise, the categorization of drinking relied on splitting it into the categories of “no current drinking” and “current drinking”. Low education was defined as the patient’s highest level of education being at or below a high school diploma. Moderate exercise was defined by 40%-59% of the reserve heart rate, during which a bit of strenuous feeling occurred with heart rate and breathing rate increasing but not becoming rapid. Body mass index (BMI) was calculated by dividing body weight (kg) by the square of the individual’s height (m²). The estimated glomerular filtration rate (eGFR) was calculated based on creatinine using the Chronic Kidney Disease Epidemiology Collaboration (CKD-EPI) 2009 equation (33). Diabetic nephropathy (DN) was diagnosed based on the urinary albumin/creatinine ratio (ACR) ≥ 30 mg/mmol or eGFR < 60 mL/min/1.73m^2^ according to the Kidney Disease Improving Global Outcomes (KDIGO) organization (34). Diabetic peripheral neuropathy (DPN) was diagnosed according to Chinese guidelines for the prevention and treatment of type 2 diabetes (35). CVD was defined as stroke and/or coronary artery disease (CAD). Dyslipidemia was defined as Cholesterol ≥ 5.2 mmol/L or low-density lipoprotein cholesterol (LDL-C) ≥ 3.4 mmol/L or Triglycerides (TG) ≥ 1.7 mmol/L or high-density lipoprotein cholesterol (HDL-C) < 1.0 mmol/L (36).

### 2.3 The diagnosis of DR

DR was diagnosed by qualified ophthalmologists based on comprehensive ophthalmologic examination, including non-mydriatic fundus photography (Optos Daytona p200T) or fundus fluorescein angiography (FFA). The diagnosis required the presence of characteristic DR lesions such as microaneurysms, hemorrhages, hard exudates, cotton-wool spots, venous beading, intraretinal microvascular abnormalities, retinal neovascularization, or photocoagulation scars, in patients with confirmed diabetes mellitus, after excluding other potential causes of retinal vascular disease. Patients without any diabetic retinal lesions were classified as non-DR (1).

### 2.4 Definition of sarcopenia

The patient was placed in the supine position. The appendicular skeletal muscle mass (ASM) was assessed using the Hologic Discovery Wi dual-energy X-ray bone densitometer (Hologic Discovery QDR Series) by certified nuclear medicine physicians. Prior to daily use, this device was calibrated using a standard calibration block supplied by the manufacturer. All scans were performed and analyzed by three fixed nuclear medicine physicians with ten years of experience, using the same software, to minimize inter-operator variability.

The appendicular skeletal muscle mass index (ASMI) was calculated by dividing ASM by the square of height (m). Low muscle mass was defined as ASMI < 7.0 kg/m^2^ for men subjects and < 5.4 kg/m^2^ for women subjects (37).

Handgrip strength was assessed with the Camry electronic grip strength meter (model EH101; Camry Scale) by trained physicians. The patient was arranged to assume either standing upright or sitting steadily, while keeping the elbow fully outstretched. The patient was instructed to perform a maximum isometric contraction with their dominant hand. Subsequently, a minimum of two measurements were taken, and the highest of these values was recorded. Low muscle strength was defined as a handgrip strength of less than 28.0 kg for men subjects and less than 18.0 kg for women subjects (37).

The 6-meter walk was assessed by measuring the time taken to cover the distance at a normal speed without slowing down, and the average result of at least two trials was recorded. Low physical performance was characterized by a walking speed of less than 1.0 m/s over a distance of 6 m (37).

According to the Consensus of the Asian Working Group for Sarcopenia (AWGS 2019). Sarcopenia was defined as having low muscle mass combined with either low muscle strength or low physical performance (37).

### 2.5 The calculation method of the inflammatory biomarkers

In this study, three inflammatory markers were used. NPAR, ALI, PIV and an additional biomarker, the neutrophil-to-lymphocyte ratio (NLR), were used to calculate ALI. All the statistics which used to calculate the inflammatory markers were measured as part of routine practice at the laboratory center in Longyan First Affiliated Hospital of Fujian Medical University. The Complete Blood Count (CBC) was calculated by the flow cytometry method. The albumin was measured by the bromocresol green method.

The formula is as follows:

NPAR: Neutrophil count/albumin (31)

NLR: Neutrophil count/lymphocyte count (38)

ALI: Body mass index × albumin/NLR (38)

PIV: Neutrophil count × platelet count × monocyte count/lymphocyte count (39)

### 2.6 Statistical analysis

Continuous variables were presented as mean ± standard deviation (SD) for normal data, or median (interquartile range, IQR) otherwise. Categorical variables were shown as frequency counts and percentages. For group comparisons, the χ² test or Fisher’s exact test was used for categorical variables, and for continuous variables, based on normality assessment, the two - sample independent t-test or Mann-Whitney U test was used.

Restricted cubic splines (RCS) were used to examine potential nonlinear relationships between each inflammatory biomarker and sarcopenia, and between each biomarker and DR. We positioned three nodes at the 10th, 50th, and 90th percentiles to construct restricted cubic spline analysis. To minimize potential bias and systematically assess the association among the three inflammatory biomarkers and sarcopenia, DR, four models were developed. The crude model was unadjusted. Model 1 was adjusted for age ≥ 60, sex. Model 2 was further additionally adjusted for low education, moderate exercise, and BMI ≥ 25kg/m^2^. Model 3 was adjusted for model 2 plus duration of diabetes, hypertension, DN, DPN, CVD, dyslipidemia, eGFR. The variance inflation factor was used to measure whether there was multicollinearity among the inflammatory markers, sarcopenia, and DR, respectively.

Mediation analysis was performed using the “mediation” package in R. The analysis examined whether NPAR, ALI, PIV served as a mediator in the association between sarcopenia and DR. Quasi-Bayesian simulation with 1000 iterations was used to estimate the mediation effects. All models were adjusted for the same set of covariates as in Model 3. No exposure-mediator interaction term was included in the models. This mediation analysis was performed under the core assumption of sequential ignorability. Given the cross-sectional design, the mediation analysis is statistical and does not imply causality.

All analyses were performed using R, version 4.0.3 software (R Foundation for Statistical Computing, Vienna, Austria). *p* < 0.05 indicated a significant difference.

## 3 Results

### 3.1 Clinical characteristics

The study included 749 patients diagnosed with type 2 diabetes. As shown in **Table 1**, the patients’ average age was 57.7 years, and 58.1% (*n* = 435) were male. The median duration of diabetes was 5 years, and the HbA1c level was 9.82% ± 2.30. The prevalence of sarcopenia was 32.6% (*n* = 244). The prevalence of DR was 25.0% (*n* = 187), 36.0% (*n* = 270) had DPN and 11.1% (*n* = 83) had DN. 10.1% (*n* = 76) had CVD and 83.7% (*n* = 621) patients had dyslipidemia.

**Table 1:** Baseline characteristics of all participants.

Variable	Total (*n =*749)	Non-DR (*n =*562)			DR (*n =*187)	*P*-value
Demographic characteristics						
Age (years)	57.7 ± 12.5		57.0 ± 12.9	59.5 ± 11.0		0.022
Age≥60, n(%)	318 (42.5)		238 (42.3)	80 (42.8)		0.986
Male, n(%)	435 (58.1)		327 (58.2)	108 (57.8)		0.986
Low education, n(%)	633 (84.9)		473 (84.6)	160 (85.6)		0.846
Moderate exercise, n(%)	135 (19.6)		97 (18.9)	38 (21.5)		0.528
BMI (kg/m^2^)	24.58 ± 3.53		24.80 ± 3.64	23.92 ± 3.06		0.003
BMI≥25 kg/m^2^, n (%)	316 (42.4)		249 (44.5)	67 (36.0)		0.053
Smoking, n (%)	191 (25.5)		147 (26.2)	44 (23.5)		0.537
Drinking, n (%)	352 (47.0)		268 (47.7)	84 (44.9)		0.567
SBP (mmHg)	134 ± 18		132 ± 17	137 ± 20		0.001
DBP (mmHg)	83 ± 12		82 ± 12	83 ± 12		0.960
Clinical condition						
Hypertension, n (%)	340 (45.4)		234 (41.6)	106 (56.7)		<0.001
Duration of Diabetes(years)	5.0 (0.3, 10.0)		4.0 (0.0, 10.0)	10.0 (5.0, 17.0)		<0.001
DN, n (%)	83 (11.1)		36 (6.4)	47 (25.1)		<0.001
DPN, n (%)	270 (36.0)		156 (27.8)	114 (61.0)		<0.001
CVD, n (%)	76 (10.1)		50 (8.9)	26 (13.9)		0.068
Dyslipidemia, n (%)	621 (83.7)		461 (82.9)	160 (86.0)		0.380
Sarcopenia, n(%)	244 (32.6)		168 (29.9)	76 (40.6)		0.009
Laboratory examination						
FBG (mmol/L)	8.69 ± 3.24		8.66 ± 3.13	8.79 ± 3.54		0.648
HBA1c (%)	9.82 ± 2.30		9.74 ± 2.29	10.06 ± 2.34		0.102
WBC, (10^9^/L)	6.94 ± 2.03		6.86 ± 1.85	7.19 ± 2.50		0.053
NEU, (10^9^/L)	4.26 ± 1.78		4.16 ± 1.60	4.53 ± 2.21		0.015
LYM, (10^9^/L)	2.07 ± 0.68		2.08 ± 0.66	2.03 ± 0.76		0.408
MON, (10^9^/L)	0.42 ± 0.15		0.42 ± 0.15	0.44 ± 0.16		0.106
Platelet, (10^9^/L)	229.82 ± 62.54		224.69 ± 58.68	245.01 ± 70.83		<0.001
Albumin, (g/L)	40.67 ± 4.20		41.04 ± 4.11	39.57 ± 4.29		<0.001
Cholesterol, (mmol/L)	5.19 ± 1.46		5.18 ± 1.47	5.23 ± 1.43		0.684
Triglycerides, (mmol/L)	1.63 (1.11, 2.63)		1.62 (1.11, 2.60)	1.71 (1.11, 2.69)		0.580
LDL-C, (mmol/L)	3.25 ± 1.00		3.24 ± 1.00	3.26 ± 1.02		0.831
HDL-C, (mmol/L)	1.11 ± 0.28		1.11 ± 0.28	1.14 ± 0.28		0.214
eGFR, (ml/min/1.73m^2^)	91.40 ± 23.17		93.83 ± 21.84	84.12 ± 25.47		<0.001
NPAR	14.58 (12.84, 16.71)		14.44 (12.66, 16.33)	15.46 (13.59, 17.60)		<0.001
ALI	52.00 (35.24, 70.18)		54.95 (36.86, 73.30)	44.74 (31.14, 60.00)		<0.001
PIV	170.04 (112.26, 275.53)		163.47 (108.70, 262.71)	210.04 (127.99, 338.33)		<0.001

BMI, body mass index; SBP, systolic blood pressure; DBP, diastolic blood pressure; DN, diabetic nephropathy; DPN, diabetic peripheral neuropathy; CAD, coronary artery disease; FBG, fasting blood glucose; HbA1c, glycosylated hemoglobin; WBC, white blood cell count; NEU, Neutrophil count; LYM, Lymphocyte count; MON, monocyte count; LDL-C, low-density lipoprotein cholesterol; HDL-C, high-density lipoprotein cholesterol; eGFR, estimated glomerular filtration rate; NPAR, neutrophil-percentage-to-albumin ratio; ALI, advanced lung cancer inflammation index; PIV, pan-immune-inflammation value.

The prevalence of sarcopenia in patients with DR (*n =* 40.6%) was higher than those without DR (*n =* 29.9%). The mean level of the white blood cell count, neutrophil count, monocyte count, platelets, cholesterol, triglycerides, NPAR and PIV in the subjects with DR was significantly higher than those without DR (*p* < 0.05). The patients with DR were older and had a longer duration of diabetes, lower BMI and eGFR compared to patients without DR (*p* < 0.05). Furthermore, compared with patients without DR, those with DR demonstrated a higher prevalence of DN, DPN and higher blood pressure (*p* < 0.05).

### 3.2 Associations among sarcopenia, inflammatory biomarkers and DR

**Figure 1:**
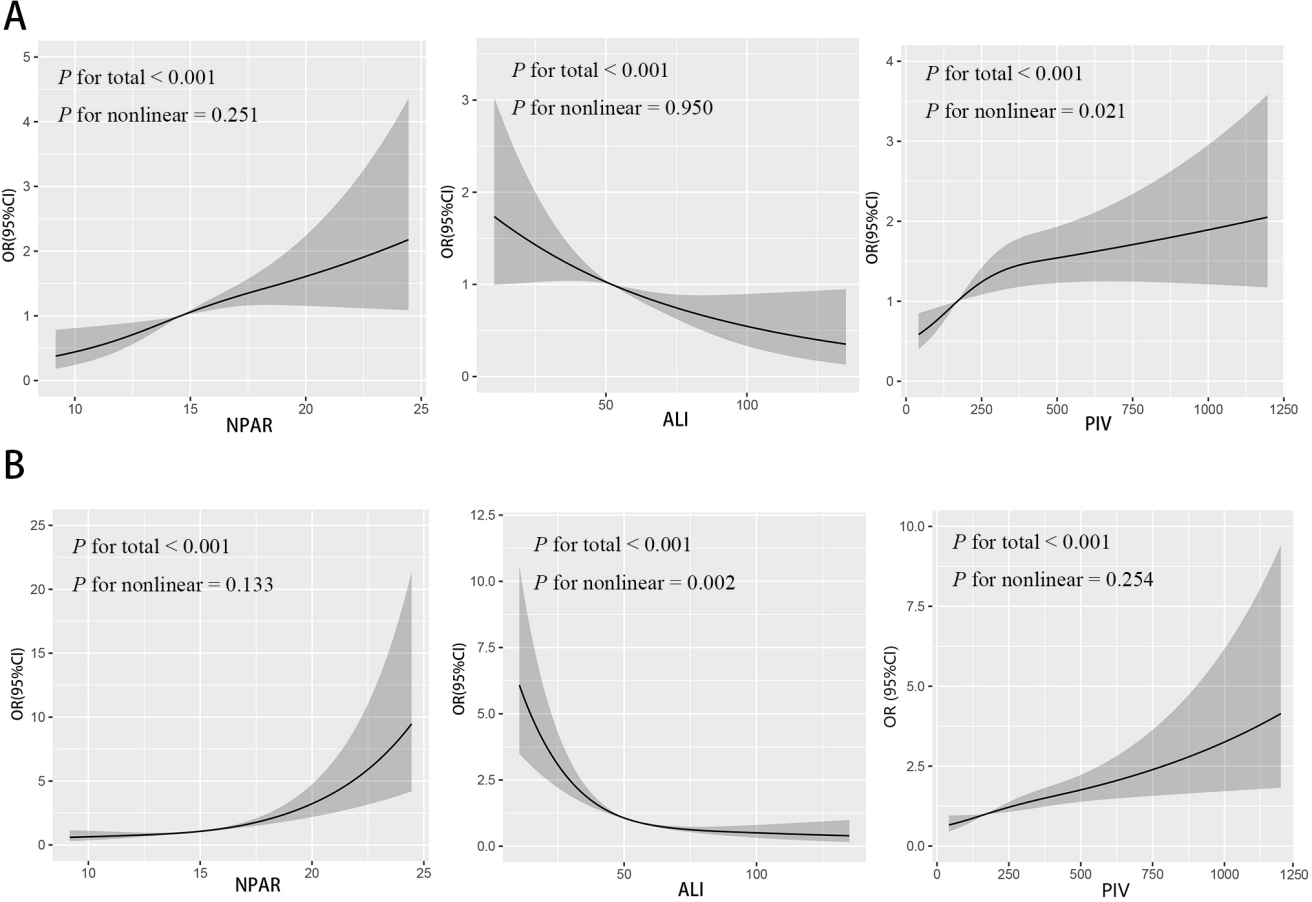
Restricted cubic spline of the inflammatory markers odds ratio of DR and sarcopenia. **(A)** The restricted cubic spline of inflammatory markers odds ratio of DR. **(B)** The restricted cubic spline of inflammatory markers odds ratio of sarcopenia. NPAR: neutrophil-percentage-to-albumin ratio; ALI: advanced lung cancer inflammation index; PIV: pan-immune-inflammation value.

As shown in **Figure 1A**, RCS analyses were used to examine the relationships between inflammatory biomarkers and DR. Both NPAR and ALI showed linear associations with DR (*p* for nonlinear > 0.05). Specifically, higher NPAR levels led to a higher DR odds, whereas higher ALI levels led to a lower DR odds. For PIV, a nonlinear association was observed (*p* for nonlinear = 0.021).As the PIV increased, the odds of DR showed an upward trend.

**Figure 1B** illustrated NPAR and PIV were linearly associated with sarcopenia (*p* for nonlinear > 0.05), such that higher NPAR or higher PIV values were linked to a higher sarcopenia odds. In contrast, ALI exhibited a nonlinear inverse association with sarcopenia (*p* for nonlinear = 0.002), meaning that sarcopenia odds showed a downward trend as ALI increased.

Associations of sarcopenia and NPAR, ALI, PIV with DR in both univariate and multivariate logistic regression models were shown in **Table 2**. In the crude model, NPAR, PIV and sarcopenia were found to be significantly positively associated with DR while ALI was negatively associated with DR.(sarcopenia:*OR* 1.59, 95% *CI* 1.14-2.26, *p* = 0.007, NPAR: *OR* 1.11, 95% *CI* 1.06-1.17, *p* < 0.001, ALI: *OR* 0.99, 95% *CI* 0.98-1.00, *p* < 0.001, PIV: *OR* 1.01, 95% *CI* 1.00-1.02, *p* = 0.005).Furthermore, the association persisted after adjusting for variables such as age≥60 years, sex, low education, moderate exercise, BMI≥25kg/m^2^, duration of diabetes, hypertension, DN, DPN, CVD, dyslipidemia and eGFR in model 3 (sarcopenia: adjusted odds ratio (*aOR*) 1.59, 95% *CI* 1.03-2.48, *p* = 0.038, NPAR:*aOR* 1.08, 95% *CI* 1.02-1.15, *p* = 0.016, ALI:*aOR* 0.99, 95% *CI* 0.98-1.00, *p* = 0.046, PIV:*aOR* 1.02, 95% *CI* 1.01-1.03, *p* = 0.044).

**Table 2:** Associations between sarcopenia, inflammation and DR in T2DM patients.

Variable	Crude		Model 1		Model 2		Model 3	
	*OR* 95% *CI*	*P*-value	*OR* 95% *CI*	*P*-value	*OR* 95% *CI*	*P*-value	*OR* 95% *CI*	*P*-value
Sarcopenia	1.61 (1.14-2.26)	0.007	1.70 (1.18-2.46)	0.004	1.63 (1.10-2.42)	0.015	1.59 (1.03-2.48)	0.038
NPAR	1.11 (1.06-1.17)	<0.001	1.12 (1.07-1.19)	<0.001	1.10 (1.04-1.17)	0.001	1.08 (1.02-1.15)	0.016
ALI	0.98 (0.97-0.99)	<0.001	0.99 (0.98-0.99)	<0.001	0.99 (0.98-1.00)	0.004	0.99 (0.98-1.00)	0.046
PIV	1.01 (1.01-1.02)	0.005	1.01 (1.01-1.02)	0.005	1.01 (1.01-1.02)	0.021	1.02 (1.01-1.03)	0.044

Model Crude: univariate logistic regression. Model 1: adjusted for age ≥ 60, sex. Model 2: model 1 plus low education, moderate exercise, and BMI ≥ 25kg/m^2^. Model 3: model 2 plus duration of diabetes, hypertension, DN, DPN, CVD, dyslipidemia, eGFR.

NPAR, neutrophil-percentage-to-albumin ratio; ALI, advanced lung cancer inflammation index; PIV, pan-immune-inflammation value; DN, diabetic nephropathy; DPN, diabetic peripheral neuropathy; CAD, coronary artery disease.

### 3.3 Relationship among inflammatory biomarkers

**Figure 2:**
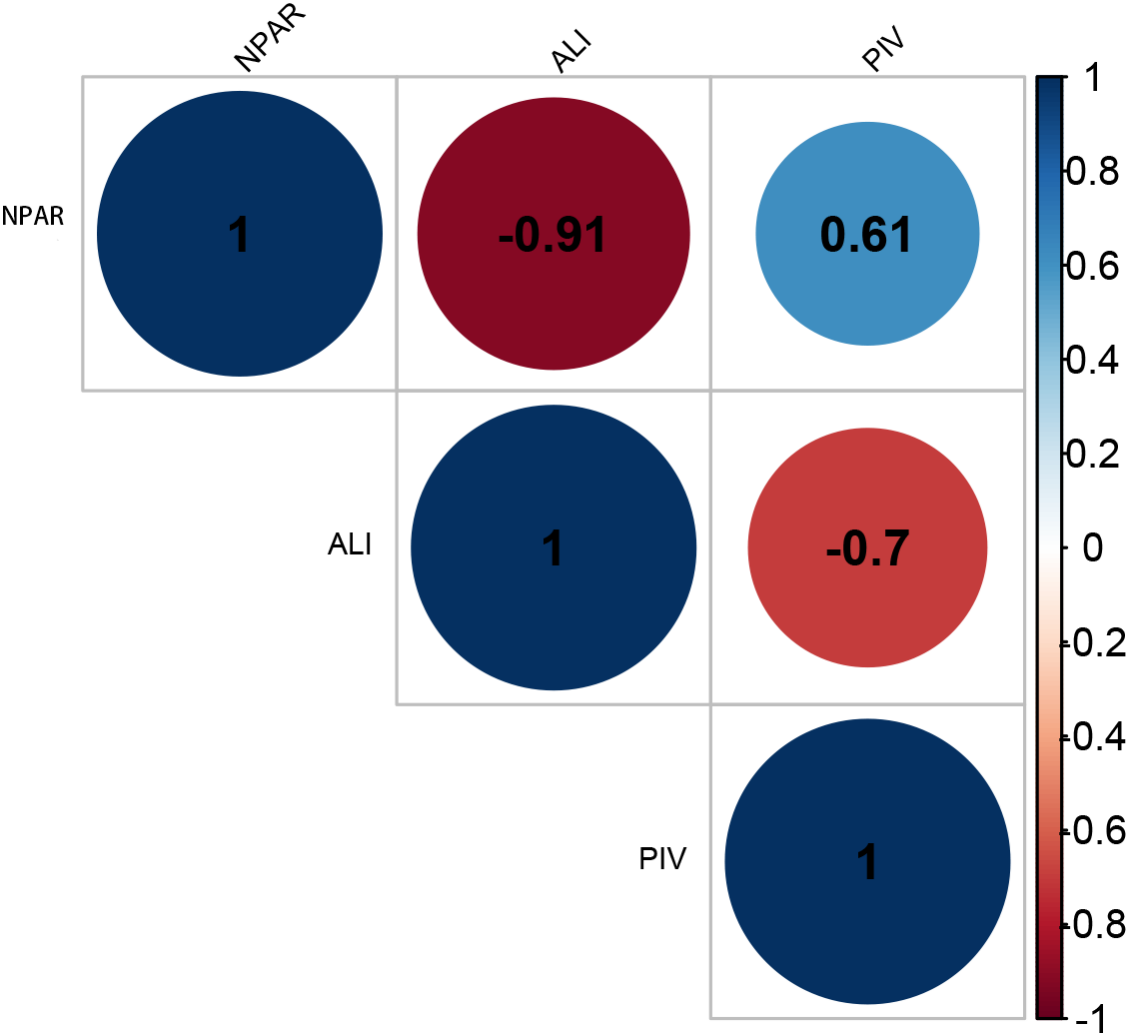
Correlations of the three blood inflammatory markers. NPAR, neutrophil-percentage-to-albumin ratio; ALI, advanced lung cancer inflammation index; PIV, pan-immune-inflammation value.

In exploring the interactions among inflammatory biomarkers, correlations among three inflammatory biomarkers were analyzed. A strong negative correlation existed between ALI and NPAR (r-value = -0.91), also existed between ALI and PIV (r-value = -0.7). Additionally, a moderate correlation was also identified between NPAR and PIV (r-value = 0.61) (**Figure 2**).

### 3.4 The mediation analysis of inflammatory biomarkers on the relationship between sarcopenia and DR

**Figure 3:**
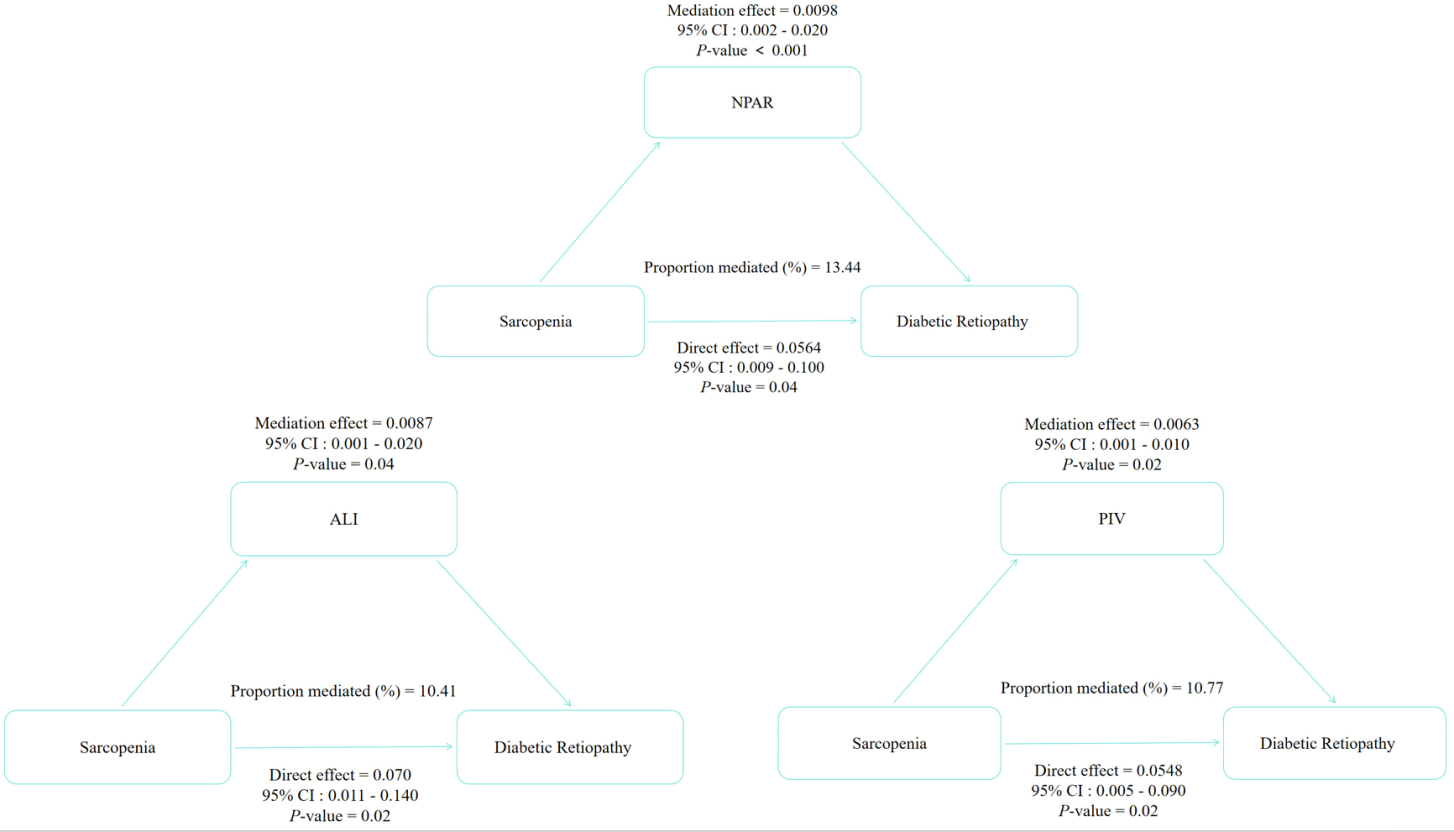
Path diagram of the mediation analysis of inflammatory markers on the relationship between sarcopenia and DR. Effect estimates are on the linear probability scale, representing the change in DR probability per one-unit increase in NPAR, ALI, or PIV. NPAR, neutrophil-percentage-to-albumin ratio; ALI, advanced lung cancer inflammation index; PIV, pan-immune-inflammation value; DR, diabetic retinopathy.

Furthermore, mediation analyses were conducted to explore the mediating effect of inflammatory markers. **Figure 3** showed the mediating role of inflammatory biomarkers in the relationship between sarcopenia and DR. Three inflammatory biomarkers statistically accounted for the association between sarcopenia and DR, with NPAR, ALI and PIV explaining 13.44%, 10.41% and 10.77% of the association, respectively (*p* < 0.05).

## 4 Discussion

Our findings demonstrated that sarcopenia was independently associated with 1.59-fold higher odds of DR after adjusting for multiple confounders, and this relationship was partially accounted for by inflammatory responses. Specifically, NPAR, ALI, and PIV accounted for 13.44%, 10.41%, and 10.77% of the indirect effects, respectively, highlighting the critical role of inflammation in bridging sarcopenia and DR.

Previous studies have explored the association between inflammation and DR by using various inflammatory biomarkers, Dascalu et al. reported significantly higher neutrophil-to-lymphocyte ratio (NLR) in DR patients. A study showed platelet-to-lymphocyte ratio (PLR) was positively correlated with the occurrence of DR and another study showed elevation of systemic immune-inflammation index (SII) increased the risk of DR (19, 40, 41). Recently, a NHANES database analysis has demonstrated a positive linear association between NPAR and DR (31). In our study, we also used NPAR to detect the relationship between inflammation and DR. It showed that as NPAR increased, the odds of DR also rose. This result was consistent with previous studies. ALI and PIV, as two novel indices, have been extensively studied in relation to chronic diseases. For instance, PIV has shown a positive association with coronary heart disease, and both indices have also been linked to dyslipidemia (42, 43). In our research, the odds of DR rose as ALI diminished or PIV escalated, indicating that a high inflammatory status was positively associated with DR. These findings contribute to the growing body of evidence on the role of inflammation in the context of diabetes, highlighting its potential involvement in the development of DR.

The relationship between sarcopenia and inflammation is bidirectional. On the one hand, sarcopenia promotes systemic inflammation through increased release of pro-inflammatory cytokines from skeletal muscle, adipose tissue, and activated immune cells. Sarcopenic muscle releases cytokines such as IL-6 and TNF-α while decreasing anti-inflammatory myokines, including irisin, thereby enhancing systemic inflammatory responses (44). On the other hand, chronic inflammation accelerates muscle protein degradation via activation of the ubiquitin–proteasome pathway and nuclear factor-κB signaling, further aggravating muscle loss (13). A recent meta-analysis demonstrated that the monocyte-to-lymphocyte ratio (MLR), systemic inflammatory response index (SIRI), and systemic immune-inflammation index (SII) are significantly elevated in individuals with sarcopenia (45). In our study, the odds of sarcopenia was significantly increased with higher NPAR and PIV, or with lower ALI, which is consistent with previous research. This further supports the evidence for a close relationship between inflammation and sarcopenia. Previous studies have consistently reported that sarcopenia is associated with a higher risk of DR. Fukuda et al. reported a 2.3-fold increased risk of DR in sarcopenic patients using a Japanese cohort (9) and similar findings were observed in a Chinese population, where sarcopenic obesity further amplified DR risk (8). Consistent with these findings, our study demonstrated that individuals with sarcopenia had significantly higher odds of developing DR compared with those without sarcopenia. it is biologically plausible that sarcopenia contribute to the development of diabetic retinopathy by reducing muscle mass, which impairs glucose metabolism, worsens systemic insulin resistance, and decreases physical activity, thereby exacerbating chronic hyperglycemia and retinal vascular stress (46). Moreover, DR-related visual impairment may result in reduced physical activity, impaired mobility, and increased sedentary behavior. Prolonged inactivity can accelerate muscle protein degradation, reduce muscle strength, and worsen insulin resistance, ultimately promoting the development or progression of sarcopenia (47, 48).

No previous study has examined whether systemic inflammation could explain the association between sarcopenia and DR. Our study demonstrated that systemic inflammation represented by NPAR, ALI, and PIV partially explained the association between sarcopenia and DR. The mediation analysis showed that NPAR, ALI and PIV accounted for 13.44%, 10.41%, and 10.77% of the indirect effects, respectively. Mechanistically, the mediation effect of NPAR may be related to the dual role of neutrophils in DR. Enhanced neutrophil activation in sarcopenic patients may exacerbate retinal vascular endothelial injury (49), Concurrent hypoalbuminemia, commonly observed in chronic inflammation, may compromise vascular integrity and promotes exudative changes in the retina (50, 51). PIV’s mediation effect may reflect activation of the platelet-monocyte axis, where increased platelet aggregation in sarcopenia releases pro-inflammatory factors and may facilitate monocyte recruitment and contribute to neurovascular unit dysfunction, ultimately promoting retinal microvascular damage and the development of diabetic retinopathy (52, 53). ALI reflects BMI, albumin, and lymphocyte counts, capturing nutritional and metabolic status. In sarcopenic patients, lower BMI and hypoalbuminemia may impair metabolic capacity and worsen insulin resistance, increasing retinal microvascular vulnerability. Higher ALI may help maintain vascular integrity and counteract chronic inflammation, thereby reducing the risk and progression of DR (54). Overall, the association between sarcopenia and DR is partially account for by NPAR, ALI, and PIV, with the remainder potentially involving other unmeasured explanation such as oxidative stress, endothelial dysfunction, growth factors, and advanced glycation end-products. These other factors may contribute to protein cross-linking and microvascular damage (1, 9, 44).

DR causes serious harm to patients’ health. Our study found that sarcopenia, NPAR, ALI, and PIV were associated with DR, and these inflammatory biomarkers statistically accounted for part of the association between sarcopenia and DR. Through consuming anti-inflammatory foods such as deep-sea fish rich in omega-3 fatty acids and doing appropriate exercise, the DR progression is delayed (55) and may potentially attenuate the impact of sarcopenia on DR, thereby reducing its detrimental effects on health. Future studies are needed to confirm our findings, evaluate the effectiveness of anti-inflammatory interventions, and verify whether these inflammatory indices can aid physicians in identifying T2DM patients who need earlier screening for DR.

However, our study has several limitations. First, due to the observational nature of the design, we cannot establish definitive causality, the mediation analysis only provides statistical estimates based on cross-sectional data. Second, the findings are derived from a Chinese adult population and may not fully represent the characteristics of other ethnic groups. Third, while we adjusted for numerous potential confounders, the possibility of unmeasured confounders cannot be entirely eliminated.

## 5 Conclusion

Sarcopenia is positively correlated with DR, with NPAR, ALI and PIV partly account for this correlation. These findings suggest that inflammation may play a role in the sarcopenia-DR relationship. Future research should include prospective studies to clarify the causal link between inflammation, sarcopenia and DR, as well as interventional and basic studies to determine whether reducing inflammation can attenuate the impact of sarcopenia on DR, and to reveal the molecular mechanism underlying the mediating effect of inflammatory.

## Data Availability

The raw data supporting the conclusions of this article will be made available by the authors, without undue reservation.
